# TOPSIS aided ensemble of CNN models for screening COVID-19 in chest X-ray images

**DOI:** 10.1038/s41598-022-18463-7

**Published:** 2022-09-14

**Authors:** Rishav Pramanik, Subhrajit Dey, Samir Malakar, Seyedali Mirjalili, Ram Sarkar

**Affiliations:** 1 Department of Computer Science and Engineering, Jadavpur University, Kolkata, 700032 India; 2 Department of Electrical Engineering, Jadavpur University, Kolkata, 700032 India; 3 Department of Computer Science, Asutosh College, Kolkata, 700026 India; 4 Centre for Artificial Intelligence Research and Optimisation, Torrens University Australia, Fortitude Valley, Brisbane, QLD 4006 Australia; 5 Yonsei Frontier Lab, Yonsei University, Seoul, Republic of Korea

**Keywords:** Computational biology and bioinformatics, Computational science

## Abstract

The novel coronavirus (COVID-19), has undoubtedly imprinted our lives with its deadly impact. Early testing with isolation of the individual is the best possible way to curb the spread of this deadly virus. Computer aided diagnosis (CAD) provides an alternative and cheap option for screening of the said virus. In this paper, we propose a convolution neural network (CNN)-based CAD method for COVID-19 and pneumonia detection from chest X-ray images. We consider three input types for three identical base classifiers. To capture maximum possible complementary features, we consider the original RGB image, Red channel image and the original image stacked with Robert's edge information. After that we develop an ensemble strategy based on the technique for order preference by similarity to an ideal solution (TOPSIS) to aggregate the outcomes of base classifiers. The overall framework, called TOPCONet, is very light in comparison with standard CNN models in terms of the number of trainable parameters required. TOPCONet achieves state-of-the-art results when evaluated on the three publicly available datasets: (1) IEEE COVID-19 dataset + Kaggle Pneumonia Dataset, (2) Kaggle Radiography dataset and (3) COVIDx.

## Introduction

COVID-19 has a colossal impact on almost every sector of society. Numerous deaths and countless positive cases are causing increased agony. Besides, governments have imposed a lockdown to confine the spread of COVID-19, which largely impacts the world’s economy and culture. More than 590 million positive cases and 6.4 million deaths due to COVID-19 alone have been recorded to date^1^. Diagnostic techniques, medicines and vaccines are some of the methods greatly researched to save humans from its disastrous consequences^2^. On the other hand, pneumonia (usually found in children) causes severe respiratory issues that occasionally lead to death. Just like any other disease, in case of both pneumonia and COVID-19, early diagnosis for detection of the same is the most essential step to not only receive proper medical attention but also curb the spread of this disease. One of the most commonly used methods for diagnosis includes a real-time reverse transcription-polymerase chain reaction (RRT-PCR) test from a nasopharyngeal swab sample. However, the major drawback of RRT-PCR is low sensitivity in detecting COVID-19 cases^3^. Moreover, these pathological diagnosis systems suffer from inter- and intra-observer variability in addition to being time-consuming.

As an alternative to pathological diagnosis, several radiological methods have been explored to diagnose COVID-19 efficiently. Computerised tomography (CT) scans, chest X-rays and magnetic resonance imaging (MRI) are some of the methods used to detect the presence of this deadly virus^4^. Computer-aided diagnosis (CAD) systems have been beneficial in easing the burden on medical professionals and ruling out the possibility of human errors. Artificial intelligence (AI) based solutions also have proven beneficial^5,6^. Notably, several research communications can be found recently that detect the presence of these viruses (COVID-19 and pneumonia) from both CT scans and chest X-ray images^7^. However, the new variants of the virus that causes COVID-19 make detection more challenging. One of the disadvantages of the reliance on radiological methods is the risk caused by radiation, which may damage the molecular structures of the human body^8^. Despite this risk factor, radiological diagnosis methods are still relied upon as they help in more accurate diagnosis than their counterparts^7,9^ do.

Convolutional neural network (CNN) aided methods have been successfully used in the past for COVID-19 and pneumonia detection^7,10^ from the chest X-ray images. The main reasons for preferring them over the duo-feature engineering and classical machine learning approaches are (1) limited need of domain knowledge for extracting learned features from input patterns, (2) capability in handling datasets with imbalanced classes^11^, and (3) the competence in handling large datasets. CNN-based approaches have been successfully explored in the domains such as speech recognition, medical image classification, handwriting recognition, human action recognition and many more. However, the most common disadvantage of such approaches is that they require high-end infrastructural support to train models properly. Moreover, CNNs are often observed to require assistance from data pre-processing to perform efficiently^12^.

Ensemble learning, in general, is the process of learning an association of multiple decision making systems. In the context of CNNs, ensemble learning methods aim to capture the complementary information of its constituent base models’ outcomes^13^. In doing so, many times researchers make use of methods having some learnable parameters. Such techniques require a substantial amount of data to train the classifier. On the other hand, non-trainable ensemble techniques do not need any training data but may require a few parameters to be tuned. For such methods to be used effectively, researchers apply some transformation operations to the raw outputs obtained from the base classifiers which is followed by an aggregation. The aggregation is either a form of hard-voting or soft-voting. In a hard-voting process, each classifier provides a binary class label (votes in favour or against). Whereas in the soft-voting process, each classifier provides a confidence score for each of the classes under consideration, based on which the final classification takes place. Multi criteria decision making (MCDM) algorithms are one of the statistical measure-based techniques used to rank the alternatives based on some given criteria. MCDMs have been extended to a large variety of works such as supplier selection strategy, benchmark machine learning tool selection for COVID-19 diagnosis, ranking of internet of things (IoT) based systems^14^ and in many more fields. Popular MCDM methods include the technique for order of preference by similarity to ideal solution (TOPSIS)^15^, vlsekriterijumska optimizacija I ompromisno resenje (VIKOR)^16^ etc.

Given the above-mentioned facts, in this work, we propose, TOPCONet, a CAD system comprising a lightweight CNN model and a TOPSIS-aided ensemble approach for diagnosis of COVID-19 and pneumonia from chest X-ray images. The lightweight deep CNN model designed here consists of only five convolutional blocks in which each block consists of four layers, namely convolutional, activation, batch normalisation and pooling layers. Each of these layers is used only once for extracting features from images. Subsequently three fully connected layers are added on top of the feature extractor i.e., after the five convolutional blocks. The CNN model is applied on three variants of input chest X-ray images, and thus we obtain three base classification models. The three image variants are: (1) the red channel image (i.e., 1-channel image), (2) normal RGB image (i.e., 3-channel image), and (3) 4-channel image generated by combining edge image obtained by applying the Robert's edge detection method. It is worth mentioning that the designed CNN-aided classifiers are trained from scratch. Finally, the decisions from the classifiers are aggregated using TOPSIS based ensemble method to obtain the final classification of an inputted chest X-ray image. The proposed TOPCONet model is evaluated on three publicly available standard datasets namely: (1) Kaggle Pneumonia + IEEE COVID dataset, and (2) Kaggle Radiography Dataset and COVIDx, and it achieves performances comparable to state-of-the-art approaches. Concisely, the highlights of the present work are summarised below.

- We develop a lightweight but efficient CNN model having much fewer trainable parameters as compared to state-of-the-art CNN models used in CAD systems for the purpose of COVID-19 and pneumonia detection.
- We design a TOPSIS, an MCDM technique, aided ensemble method which is found to be more effective than standard ensemble methods such as majority voting, product rule and sum rule.
- The performance of the proposed model on three public datasets is comparable to state-of-the-art methods that require heavy computational resources.

The remaining parts of the paper are organised in the following manner. In the “Literature review” section, we first briefly review some past works from the literature of COVID-19 and pneumonia diagnosis system and then analyse each of them to describe their advantages and shortcomings. In the “Preliminaries” section, we briefly describe some prerequisites useful in describing the working procedure of the proposed model. Next, in the “Proposed method” section, we describe in detail how our model (i.e., TOPCONet) works. In the “Experimental results” section, we present all the experimental findings related to this work while we analyse the results and explain each of the experimental findings in the “Discussion” section. We also summarise the advantages and limitations of our work in this section. In the “Conclusion” section, we end with concluding remarks and some scopes for future research.

## Literature review

Several research attempts were made by researchers to design effective CAD systems for COVID-19. Here, we review some of the existing works employed for early detection of COVID-19 and/or pneumonia from chest X-ray images. Wang et al.^17^ designed a CNN architecture, dubbed as COVID-Net, that achieved 80% sensitivity for COVID-19 detection. This is one of the fundamental works that laid the ground-work for further research in this domain with deep learning-based CAD models for detection of the said diseases. The article by Demir^12^ presented a long short term memory (LSTM) network-based approach where the Sobel edge detection and marker-controlled watershed segmentation approach were used in the pre-processing stage. The author was able to significantly boost the model performance, but the computational cost was considerably high because of training both CNN and LSTM models. Convolutional LSTM (ConvLSTM) based approach has also been seen in the literature recently^18,19^.

Classical handcrafted feature engineering-based approaches have also proven their competence for COVID-19 detection. Panetta et al.^20^ proposed a novel shape-dependent Fibonacci-p patterns-based feature descriptor for COVID-19 detection. In another work, Chandra et al.^21^ first extracted the gray-level cooccurrence matrix (GLCM) and histogram oriented gradient (HOG) based features and then used the binary grey wolf optimizer (BGWO) based feature selection technique to select the near-optimal feature set. Finally, a bi-stage majority voting-aided ensemble mechanism was utilised with the seven different machine learning-based classifiers. The authors classifies normal and abnormal images quite successfully, but the model performance was not good while classifying infected samples into pneumonia and COVID-19 cases. One of the possible reasons for such a behaviour might be related to a recent medical research findings^22^ where the authors state “In some cases, definite discrimination of the two (COVID-19 and pneumonia) entities might be impossible solely based on the imaging, however, some radiologic features may suggest one diagnosis over the other”.

Oh et al.^23^ used a patch-based classification strategy to mitigate the difficulties due to a limited training sample while using a CNN model-based classification strategy. They first segmented the images using deep CNNs and then generated random patches to train the CNNs using the ResNet-18 model. Thus they were able to train the CNN model using limited training datasets. The approach also lowered the training cost by a margin. In the said work, the normalised validation accuracies were used as fuzzy measures for the base classifiers but it may not work with other datasets. Similarly, in a very recent article, Paul et al.^24^ proposed to use an inverted bell-shaped weighted average scheme to ensemble the probabilistic outcomes of deep transfer learning models. In another work, Das et al.^25^ designed a bi-stage CAD system for the detection of COVID-19 and pneumonia-infected chest X-ray images. In the first stage, the authors segregated the infected chest X-ray images from the normal ones, and in the later stage, the COVID-19 cases were identified from the infected cases. The authors used the pre-trained VGG-19 as the feature extractor, and a shallow learner was employed as the classifier. The performance of the bi-stage model was good but once a sample is classified erroneously in the first stage, it remained unclassified throughout. Moreover, the bi-stage classification scheme for detecting a COVID-19 or pneumonia case needs classification of a sample twice, which lengthens the testing time. A stacked CNN model using sub-models of VGG-19 and XceptionNet was proposed by Gour et al.^26^, but use of such large CNN models like VGG-19 and XceptionNet increases the training and inference time. Hasoon et al.^27^ used a combination of image preprocessing techniques and machine learning algorithms to get 6 different models those are local binary pattern (LBP)-kNN, HOG-kNN, Haralick-kNN, LBP-SVM, HOG-SVM, and Haralick-SVM for chest X-ray image classification. The authors showed that the use of machine learning algorithms failed to produce better result over the CNN based models. A new COVID-19 detection model CoWarriorNet was proposed by Roy et al.^28^ that consists of two networks a classification network and a confidence network. The proposed architecture is a derivative of U-Net architecture, which results in poor extraction of image-derived information.

Ouchicha et al.^29^ provided a CNN architecture with a sort of inter-network skip connections for screening of COVID-19 cases from chest X-ray images. The authors used two parallel CNN models and connected each of the layers of networks with inter-network and intra-network residual connections for feature transfer to encounter the vanishing gradient problem. Additionally, it is interesting to note that downsampling and skip connections have also been proposed in the literature in the past for COVID-19 detection^30^. Khuzani et al.^31^ employed multiple feature extraction schemes in which a novel pooling strategy was used to extract only the relevant features. Finally, the authors used a fully connected neural network to classify chest X-ray images using the extracted features. The authors used the machine learning-based approach, instead of the conventional CNN approach which could significantly reduce the computational cost, but the approach failed to tackle the class imbalance issue. In another work, Kenaway et al.^32^ first used a popular CNN architecture as a feature extractor and then proposed an advanced version of the squirrel search optimisation algorithm to select the best set of features. They finally used the multi-layer perceptron (MLP) on top of the selected features for classification, leading to a significant increase in training time, as the transfer learning model is fine-tuned. Besides, the feature selection approach is trainable and the classifier also needs time to train. Gour et al.^33^ introduced a uncertainty aware CNN model known as the UA-ConvNet model. The problem of this CNN model is that in its initial stage, a pre-trained EfficientNet-B3 model is fine-tuned, which makes it time-taking. Bashar et al.^34^ used transfer learning concept while using CNN models like AlexNet, GoogleNet, VGG-16, VGG-19 and DenseNet to classify chest X-ray images. The authors enhanced the input image quality using techniques like anisotropic diffusion filter, Fourier transform, and edge-aware local contrast manipulation. Here, again the use of transfer learning techniques makes the process computationally expensive to train. Similar problem faced by Senan et al.^35^, where ResNet50 and AlexNet models were used to generate features which are combined with GLCM and LBP features.

Khan et al.^36^ proposed an XceptionNet-based classification model, which performed well, but the metrics reveal that it somewhat failed to handle imbalanced classes despite using a CNN-based architecture. Hussain et al.^37^ proposed a 22-layers deep CNN model. Goel et al.^38^ proposed a CNN model whose hyperparameters were optimised using a nature-inspired meta-heuristic approach: BGWO. This method provided a good alternative to the tiring grid search method, but the model has a slightly higher false-positive rate, which might not be useful in practical field. Aslan et al.^39^ designed a hybrid approach using a modified version of the AlexNet hybridised with bi-directional LSTM (BiLSTM) providing better results worth-while results in processing the features for classification. Multi-level feature extraction technique was used by Naeem et al.^40^ where the authors first extracted features like GIST, scale invariant feature transform (SIFT), and CNN based deep features and then they used LSTM network to perform classification. Similarly, classical classifiers like artificial neural network (ANN), SVM, KNN, and deep learning classifiers like recurrent neural network (RNN), LSTM were used for predicting COVID-19 cases from chest X-ray images by Goyal et al.^41^. The problem with LSTM and RNN architectures are that they are prone to over-fitting and difficult to apply dropout algorithm there.

## Motivation

Numerous deep learning-based methods have been explored in the past for designing CAD systems for the diagnosis of COVID-19 and pneumonia from chest X-ray images. Most of them aimed at designing a system that could perform more efficiently than its predecessors, which is the primary objective of designing a new CAD system at its initial stage. Hence, in many cases, these models became computationally heavier. However, an ideal CAD system should be computationally inexpensive and memory-efficient for its wide applicability and portability so that such a model can be easily executable in a resource-constrained environment without compromising its performance. Besides, the increment of learnable parameters in CNN models increases the space and time requirements. Another important issue is that such models at times extract some redundant features which in turn consume additional space and time during execution without much improvement of performance^42^. Additionally, according to the lottery ticket hypothesis^43^, we can prune the learnable parameters of a heavy CNN model to make it much more cost-efficient and portable without compromising its performance. Keeping these facts in mind, we aim at design a lightweight CNN model that can detect COVID-19 and pneumonia-infected chest X-ray images from normal ones. Additionally, it is to be noted that the authors of the works^6,21,44^ used a hierarchical approach to design better CAD models for diagnosis of the said diseases, in which they applied various heavier CNN architectures at each stage. The use of multiple pre-trained CNN models along with evaluating an input image several times makes these models inefficient in terms of storage and computation needs. Hence, we evaluate an input image only once to make it more memory and time-efficient. Apart from these, some methods^6,45^ used ensemble techniques in which different CNN models were applied as base classifiers to obtain different learned features from input images. However, the study^46^ shows that we can gather complementary information by pre-processing the original image to extract features. Hence in our model, we pass two differently pre-processed images along with the original one to the CNN model for extracting three different features from it. We also observe from the literature that the work reported by Paul et al.^45^ used a machine learning-based ensemble technique and showed its effectiveness in improving the end performance. In a work by Pramanik et al.^6^, where the authors propose a fuzzy aggregator to ensemble the CNN outcomes which is non-trainable in nature.

In this work, to capture the complementary information provided by the base classifiers, we propose an ensemble with an MCDM-based aggregator known as TOPSIS. The ensemble framework has a few tunable parameters compared to the machine learning-based ensemble techniques. The use of such methods aims at developing a lightweight but effective CAD model for distinguishing COVID-19 and pneumonia-infected chest X-ray images from normal ones.

## Preliminaries

### Robert's edge detection technique

The Robert's edge detection technique uses 2D spatial gradient calculation to detect edges in an input image. It uses two operators of size $2\times 2$, known as Robert cross operators, which help in calculating the gradient image. The gradient image is used for identifying the edges in the image. Here, our idea is to extract the minute details present in a chest X-ray image and thus use a small-sized mask while calculating gradient image, which would be helpful rather than using larger masks. This is why we chose this edge detection technique. The Robert cross operators are shown in Eqs. (1) and (2).

1 $$\begin{aligned} {\delta _{x}}= & {} \begin{bmatrix} +1 &{} 0\\ 0 &{} -1 \end{bmatrix} \end{aligned}$$

2 $$\begin{aligned} {\delta _{y}}= & {} \begin{bmatrix} 0 &{} +1\\ -1 &{} 0 \end{bmatrix} \end{aligned}$$

In this edge detection technique, an input image is convolved with these two operators i.e., $\delta _x$ and $\delta _y$. Let $\delta _x$ and $\delta _y$ be convolved with a gray-scale image (say, $I_g$) and generate two gradient images (say, $G_x$ and $G_y$) using Eqs. (3) and (4).

3 $$\begin{aligned} G_x= & {} I_g*\delta _x \end{aligned}$$

4 $$\begin{aligned} G_y= & {} I_g*\delta _y \end{aligned}$$

In Eqs. (3) and (4), $'*'$ is the convolutional operator. The magnitude image corresponding to the gradient images (say, *G*) is calculated using Eq. (5).

5 $$\begin{aligned} G = \sqrt{G_x^2 + G_y^2} \end{aligned}$$

We obtain the final edge image using a threshold value (say, *th*). In the present work, we use the mean of the gradient magnitude values appearing in *G*. The parameter *th* is calculated using Eq. (6).

6 $$\begin{aligned} th = \frac{1}{MN} \sum _{x=1}^{M} \sum _{y=1}^{N} G(x,y) \end{aligned}$$

In Eq. (6), *M* and *N* stand for the height and width of the input image respectively. Finally the edge image (say, $I_e$) is obtained using Eq. (7).

7 $$\begin{aligned} I_e(x,y) = \left\{ \begin{array}{lr} edge \ pixel &{} : G(x,y) < th\\ background \ pixel &{} : otherwise \end{array} \right. \end{aligned}$$

### Convolutional neural network

This is probably one of the most revolutionary research findings in the domain of document image processing^47^, which later extended to multiple fields such as video processing^48^, speech processing and emotion recognition. CNN is used as a discriminative architecture^49^ inspired by the concept of a time delay neural network (TDNN). In TDNN, the weights are shared in a temporal dimension, which ultimately reduces computation. On the other hand, the CNN model does not require weights to be shared, and convolutions are replaced by matrix multiplication as in standard NNs. This replacement significantly decreases number of weights. Moreover images can be fed as raw input to the network, whereas in classical NN models, features, extracted from the images, are fed to the NNs. In other words, CNNs are seen as a combination of a learned feature extractor and a classifier. Weights for both the classifier and the feature extractor model are learned using the back propagation algorithm^47^. Some important parts/characteristics of any CNN models are described in the later parts.

#### Convolutional layer

One of the most important parts of any CNN model is the convolutional layer which is suggested by its name. In a convolutional layer, a linear operation occurs in which the input image is convolved with a set of learnable weights. For applying the convolutional process on an image, a two-dimensional array (i.e., matrix) of weights is used which is known as the kernel or filter. The filter is smaller in size compared to the input image. To perform convolution element-wise matrix multiplication is performed between the filter matrix and a filter-sized patch of the input image. After this, all the values of the patch are added together to produce a single value. By using a filter smaller than the input size it can then be applied to the various parts of the input during convolution. Thus, the same weights can be applied to the various parts of the input data resulting in translation invariance. The invariance to translation is an important property for knowing whether a feature is present in the input image rather than in the location of the feature in the image.

#### Activation function

Activation functions play a crucial role during the training of the CNN models. Let $z_i=g(x,w_i)$ be the mathematical formulation of any neuron and $y_i=f(z_i)$ be the value that is to be transmitted to the next neuron. The use of an activation function is popular because it helps in countering the vanishing gradient problem associated with any CNN model. In standard NNs, activation functions control the flow of information among the neurons present therein. There are several diverse activation functions in literature at present. The choice of activation functions mainly depends on the objective of the work at hand. Probably, one of the most popularly used activation functions is the rectified linear unit (ReLU) which is well used for image recognition-based tasks^47^.

#### Batch normalisation

Initially suggested by Ioffe et al.^50^, batch normalisation greatly improvises the convergence of the CNN model during training and also helps produce stable results. Batch normalisation helps in coordinating the update of weights in multiple layers in the model. To do so, it scales the output of the layer such as standardising the activations of each input variable or the activations of the nodes of the previous layer. Here, standardising means rescaling data to have a mean of 0 and a standard deviation of 1. This standardisation stabilises during the training phase of the CNN model and also speeds up the training time.

#### Pooling layer

A pooling layer is designed to reduce the dimension of feature maps. This layer ensures that the relevant information passes on to the next level of the network while the redundant features are discarded. Thereby pooling serves as a filter mechanism for the extracted features. In this work, we use max-pooling which returns the maximum local value of each pooling window.

#### Fully connected network

**Figure 1:**
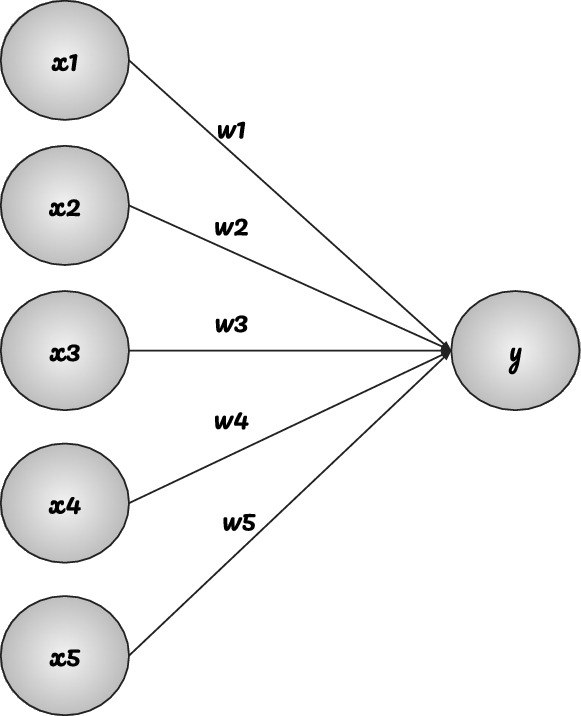
A pictorial representation of how a neuron of one layer connects with neurons of other layer.

**Figure 2:**
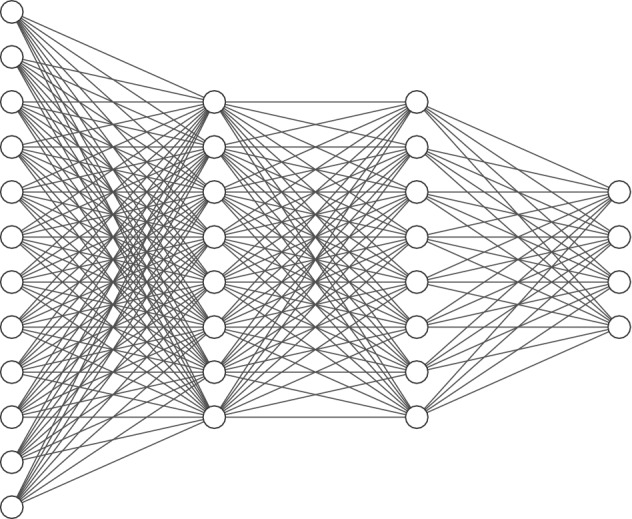
A pictorial description of classical feed-forward neural network with one input layer of size 12, two hidden layers each of size 8 and an output vector of length 4. This figure has been generated using a tool developed by Alex LeNail https://alexlenail.me/NN-SVG/.

In fully connected network (FCN) layers, the neurons of the current layer remain connected to each neuron of the previous layer. Figure 1 shows how a single neuron of one layer connects with multiple neurons of another layer during the construction of a multi-layered FCN model. In this figure, the node *y* is represented with the weighted sum of its inputs as shown in Eq. (8). In this equation, the parameters *w* and *b* are optimised using the back propagation algorithm. Relevant studies in the past have shown that a neuron having a larger *w* value means that it is more important. The parameter *b* is the bias variable used to shift the value of *y*. The output layer of the FCN part of a CNN model is an N-dimensional vector, where N is the number of classes for a classification problem. A pictorial description of the classical multi-layered FCN model is given in Fig. 2.

8 $$\begin{aligned} y=\begin{bmatrix}w1&w2&w3&w4&w5\end{bmatrix} \cdot \begin{bmatrix} x1\\ x2\\ x3\\ x4\\ x5\\ \end{bmatrix}+b \end{aligned}$$

The final FCN layer uses a softmax function (see Eq. 9) that determines the class level probabilities of an input for a classification problem.

9 $$\begin{aligned} softmax(z_j)=\frac{e^{z_j}}{\sum _{x=1}^{N}e^{z_x}} \end{aligned}$$

In Eq. (9), $z=[z_1,z_2,...,z_N]$ is the input to the softmax function.

### TOPSIS algorithm

TOPSIS, proposed by Hwang and Yoon^15^, is a simple (though effective) multi-criteria decision analysis (MCDA) or MCDM method. It is derived from the idea that the geometric distance of the highest-ranked alternative is the nearest to the ideal solution while the lowest-ranked alternative is the nearest to the worst solution. The positive ideal solution capitalises on the beneficial attributes most while minimising on the cost attributes. The TOPSIS method uses all of the information in the form of a decision matrix and criteria weights as a vector and generates a ranking for all the alternative candidate. A detailed procedure is described in Algorithm 1.

**Figure:**
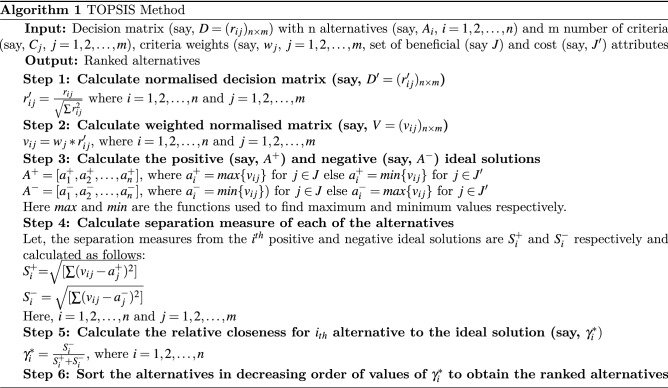


## Proposed method

**Figure 3:**
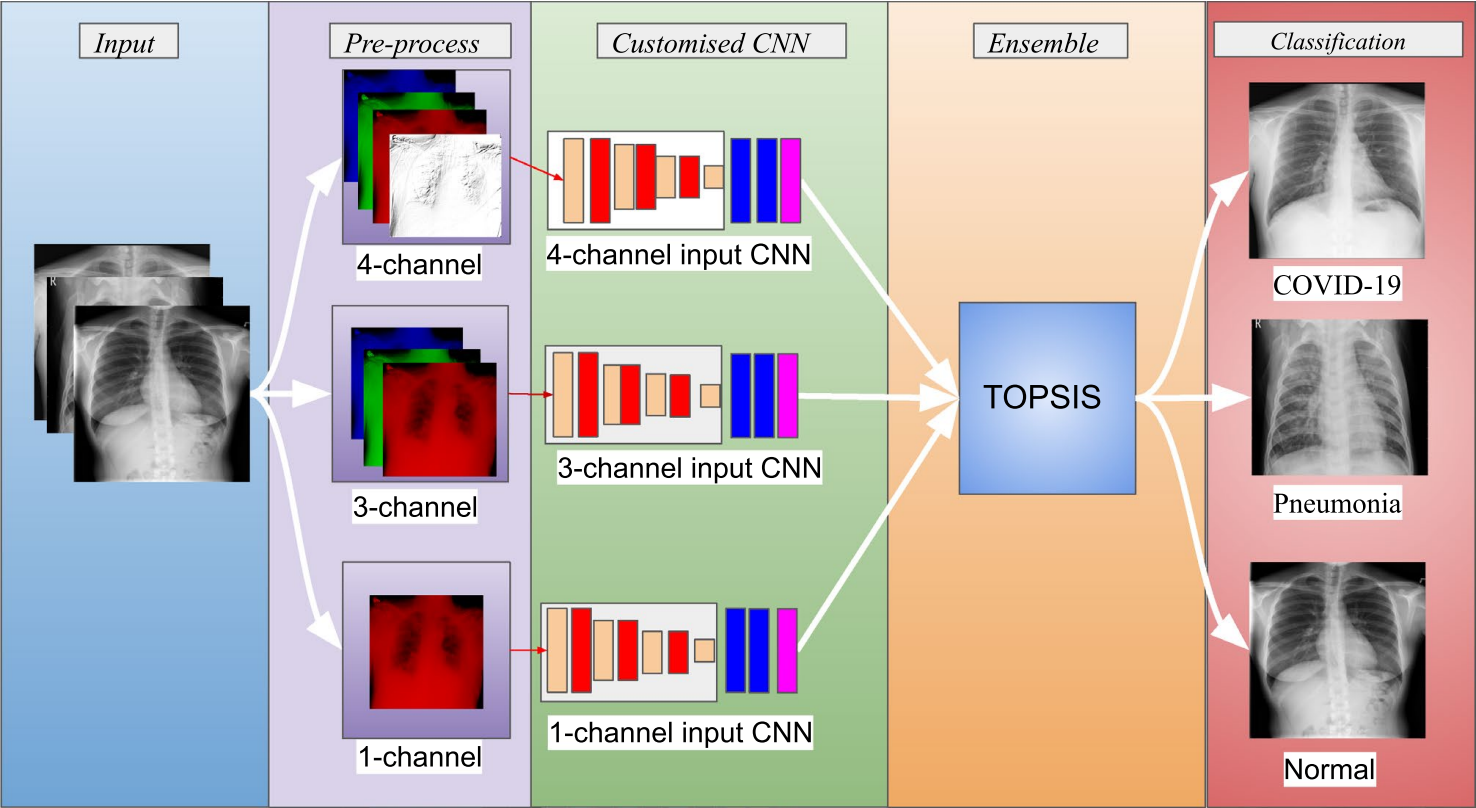
The pipeline for the proposed model. Here, the CNN architecture for all three base classifiers is the same. Images are taken from the public datasets found in^51,52^.

In this work, we propose TOPCONet, an ensemble-based approach using lightweight CNN based three base learners (hereafter termed Classifier 1, Classifier 2 and Classifier 3) to detect COVID-19 and pneumonia from chest X-ray images. The base classifiers use the same CNN architecture but work with different forms of input images. The image variants are red channel image (i.e., 1-channel red image), original RGB image, and a 4-channel image prepared by combining the edge image generated by the Robert's edge detection method with the original RGB image used in Classifier 1, Classifier 2 and Classifier 3 respectively. To begin with we resize all the images to the spatial dimension of $224\times 224$ pixels to feed to the base classifiers. While evaluating we estimate the class probability scores using the base classifiers and feed them to the TOPSIS-based ensemble method. The complete pipeline for our proposed model can be found in Fig. 3.

### Customised CNN

**Figure 4:**
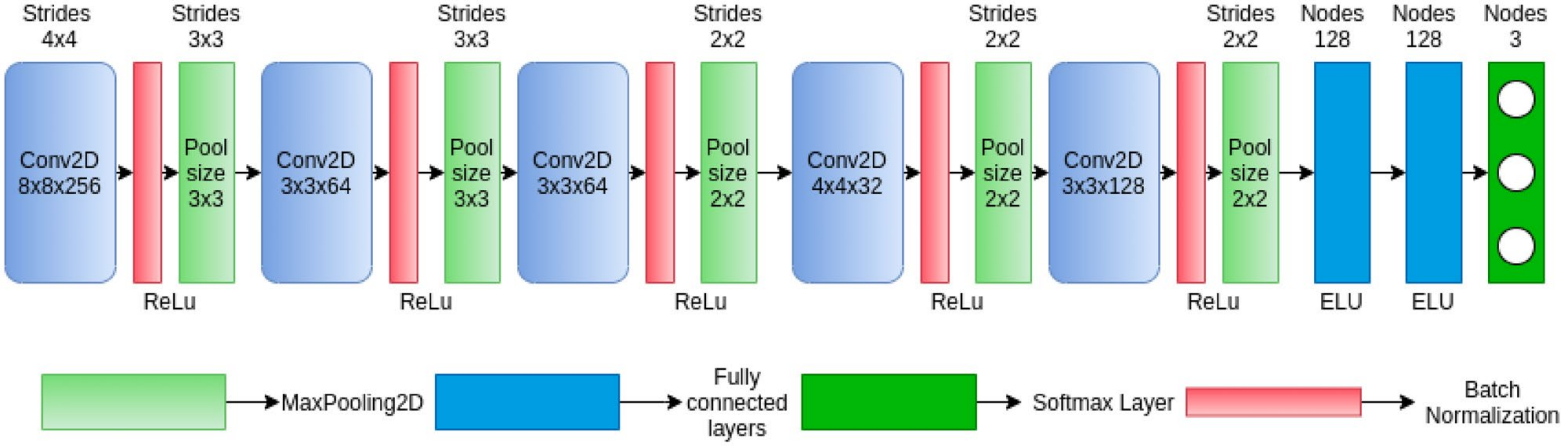
Architecture of the proposed CNN model.

It has been described earlier that a CNN architecture consists of two main components viz., a feature extractor and a classifier network. Therefore, while designing a customised CNN architecture for any specific task, both the feature extractor and the classifier should be stressed upon. A very deep network consisting of many layers might be effective for extracting very deep features but on the other hand, optimisation of the model becomes a difficult task which means the optimisation of the very deep network is much difficult when compared to small-sized networks. The same has been empirically and theoretically shown by Choromanska et al.^53^. The major motivations for using CNNs as a feature extractor over handcrafted features is the presence of shared weights, pooling, local connections and many possible layers^47^. Therefore for each of these layers, while designing, one thing that should be considered is the extraction of the best possible features for the classifier to perform efficiently. It must also be kept in mind to minimise the number of redundant features. To efficiently deal with this, nowadays deep feature selection-based approaches are also gaining popularity^42,54^. Filter pruning strategy is one of the appproaches to reducing the number of redundant features; one such work was communicated by Luo et al.^55^. On a similar note, in this work, we aim to extract meaningful and less redundant features. Considering the mentioned facts, the customised CNN designed with five blocks of convolutions, is carefully tuned to extract the best possible features and minimize the redundant extracted features. Each level of convolution is followed by a layer of activation to control the flow of information to the next level of convolution and for, batch normalisation for re-scaling and re-centring of the values to have smoother learning and max-pooling to get rid of redundant features. It the end, an FCN with two hidden layers is coupled with exponential linear unit (ELU) activation function to impart a non-linearity to the input signal. Thus, we ensure that both the feature extractor and the classifier work efficiently. The detailed structure is shown in Fig. 4.

### TOPSIS-aided ensemble method

As already been mentioned, in the present work, we employ a TOPSIS-aided ensemble method to decide the final class of an input chest X-ray image depending on the class level confidence scores generated by the base classifiers. We use the available classes as the alternatives and the confidence scores from each of the base classifiers as the criteria values for each of the alternatives (i.e., classes). The *ij*th element of the decision matrix (i.e., $r_{ij}$) used in the TOPSIS method is constructed by considering the confidence score of the *i*th base classifier corresponding to the *j*th class. We employ all the steps as described in Algorithm 1 on the decision matrix to obtain the top most-ranked alternative which here is the final class. It should be noted that we resort to column normalisation of the decision matrix to comply with the TOPSIS algorithm. During the normalisation process, we divide the input score by the square root of the summation of squares of the observations. In our case, all the criteria are beneficial.

#### Computational complexity of the TOPSIS aided ensemble model

As the proposed ensemble method is non-trainable, it is important to discuss the computational complexity of the model. In this part, we discuss the computational complexity using the ${\mathscr {O}}$-notation. The ensemble method requires constant number of operations for each of the samples. For each of *n* base classifiers and *m* alternatives (i.e., number of classes), normalisation and weight assignment. The complexity of the model is ${\mathscr {O}} (n\times m)$. The complexity while determining the positive and negative ideal solution and distance from the two is ${\mathscr {O}} (n\times m)$. Furthermore, the complexity of finding the top ranked alternative is ${\mathscr {O}}(m)$.

### Statement of ethical approval

All procedures performed in studies involving human participants were in accordance with the ethical standards of the institutional and/or national research committee and with the 1964 Helsinki Declaration and its later amendments.

## Experimental results

In this section, we discuss and also attempt to explain the experimental findings. It should be noted that all the performances are evaluated on machines supported by Nvidia Tesla T4 GPUs. The programming environment used to train and test our model is Python 3.8.

### Dataset description

To evaluate the performance of our proposed method for COVID-19 and pneumonia detection from chest X-ray images we use samples from two datasets. The first dataset (termed as Dataset-1 hereafter) consists of chest X-ray images of pneumonia-affected patients and normal subjects from Guangzhou Medical Centre: Chest-X-ray-Pneumonia^52^ and COVID-19 affected patients from IEEE COVID-chest X-ray-dataset^51^. The second dataset, termed here after as Dataset-2, is hosted publicly on Kaggle and is popularly known as the COVID-19 Radiography Database^56^. Dataset-2 has been created in collaboration of researchers from Qatar University (Qatar), University of Dhaka (Bangladesh), Pakistan and Malaysia. The dataset consists of chest X-ray images of COVID-19 positive, normal and pneumonia-infected cases. The distributions (i.e., number of samples in train and test set) of both the datasets have been shown in Table 1. It is to be mentioned here that during model training we have randomly selected 15% of the training samples as validation samples.

**Table 1:** Distribution of datasets for training and evaluating the present TOPCONet model.

Dataset	Source	Train samples			Test samples		
		COVID-19	Normal	Pneumonia	COVID-19	Normal	Pneumonia
Dataset-1	Kaggle Pneumonia^52^ and IEEE COVID-19^51^	739	1072	3100	185	269	775
Dataset-2	KaggleCOVID-19 Radiography Database^56^	2893	8154	1076	723	2038	269

### Tuning the hyperparameters of customised CNN model

**Figure 5:**
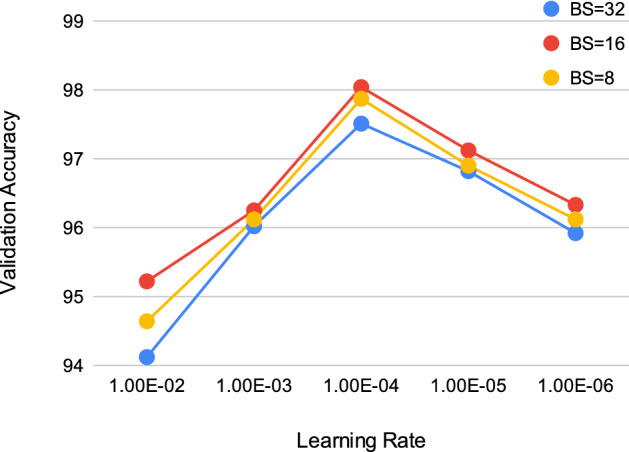
Validation accuracy (in %) with respect to different learning rates used to train the customised CNN model.

In typical CNN models, hyperparameter tuning is a very essential and tedious job. Hyperparameters of a deep learning model to an extent indicate the learning capability of such a model. It is essential to set two main hyperparameters, namely batch size and learning rate to obtain near-optimal model performance. The batch size controls the number of samples to be loaded for training at one go while the learning rate determines the learning capability of the model which says to what extent the newly acquired information overrides the previous. Here we estimate the hyperparameters using samples of Dataset-1 and propagate these parameters to train the model for Dataset-2. Here we would also like to mention that we tune the said hyperparameters only for Classifier 2 as it takes the standard RGB images as input. For tuning the hyperparameters, we randomly select 15% of the training dataset as the validation samples. For this, we employ the grid search method to select the near-optimal batch size and the learning rate. The learning rate is selected from the set $\{1e-02,1e-03,1e-04,1e-05,1e-06\}$ while for selecting batch size we use the set $\{8,16,32\}$. From Fig. 5 it is observed that the validation accuracies converge to their optimum solution for learning rate = $1{\text {e}}-4$ and batch size = 16. For smooth learning we rely on the popularly used Adam optimiser and categorical cross-entropy as the loss function.

### Performance comparison: customised CNN model vs. state-of-the-art CNN models

It has already been mentioned that we propose a customised CNN model that is lightweight in nature (see Fig. 4). Based on this CNN model, three different classifiers are formed. We compare the performances of the proposed classifiers (including TOPCONet) with state-of-the-art CNN models following the transfer learning concept and training from the scratch. In the transfer learning approach, we load the pre-trained weights of the respective CNN models which are obtained after training the models on the ImageNet dataset. Subsequently, using the loaded weights we fine-tune the models on both datasets. To maintain uniformity during performance comparison, the total number of epochs used is 15 for both state-of-the-art CNN models and the proposed classifiers. All the results are evaluated on test samples of the said datasets i.e., following hold-out test set samples approach. The comparative results are presented in Tables 2 and 3 for Dataset-1 and Dataset-2 respectively.

**Table 2:** Performance comparison of the proposed 3 base classifiers and TOPCONet with some state-of-the-art CNN models utilising the concept of transfer learning and training from scratch on Dataset-1.

Model	# Trainable parameters	Performance (in %) in terms of				Total time (in s)	
		Pre	Rec	F1-score	RA	Train	Test
SqueezeNet	1,248,424	93.12	93.27	93.19	93.32	112.6	2.4
SqueezeNet*	1,248,424	92.24	92.04	92.14	92.35	226.3	4.5
MobileNetV2	3,504,872	98.23	98.00	98.11	97.96	102.1	3.1
MobileNetV2*	3,504,872	98.39	97.65	98.25	98.12	448.4	4.5
ResNet101	44,549,160	94.33	91.00	92.67	94.38	331.5	7.0
ResNet101*	44,549,160	96.83	92.70	94.41	95.03	779.2	6.8
DenseNet121	7,978,856	97.67	96.67	96.67	97.96	184.9	6.0
DenseNet121*	7,978,856	97.57	98.12	97.84	97.80	637.5	5.7
VGG-19	143,667,240	96.67	96.33	96.67	98.20	207.0	11.1
VGG-19*	143,667,240	95.14	97.53	96.21	96.41	625.6	8.3
InceptionV3	27,161,264	96.33	96.67	96.67	96.69	209.5	4.7
InceptionV3*	27,161,264	95.5	96.43	95.94	96.58	430.9	7.6
Classifier 1^*^	**306,467**	97.34	97.00	97.67	97.96	**55.3**	**0.9**
Classifier 2^*^	339,235	97.67	97.34	97.67	98.04	68.4	1.5
Classifier 3*	355,619	98.34	98.00	98.37	98.53	86.2	2.4
TOPCONet	1,001,324	**98.47**	**98.26**	**98.37**	**98.78**	N/A^#^	4.9

For citing recall (Rec), precision (Pre) and F1-score micro-average method is used.

*After the CNN models’ name means that the models have been trained from scratch.  ^#^As the proposed ensemble method is non trainable, hence time required to run on the training split is not applicable (N/A).

RA indicates recognition accuracy and bold faced numbers indicate the best scores.

**Table 3:** Performance comparison of the proposed 3 base classifiers and TOPCONet with some state-of-the-art CNN models utilising the concept of transfer learning and training from scratch on Dataset-2.

Model	# Trainable parameters	Performance (in %) in terms of				Total time (in s)	
		Pre	Rec	F1-score	RA	Train	Test
SqueezeNet	1,248,424	95.35	95.20	95.27	95.32	165.4	5.5
SqueezeNet*	1,248,424	94.56	94.23	94.39	94.78	556.6	4.9
MobileNetV2	3,504,872	95.96	95.88	95.92	96.14	270.5	5.0
MobileNetV2*	3,504,872	95.93	97.26	96.57	97.6	1047.0	5.2
ResNet101	44,549,160	95.20	95.45	95.32	95.48	693.3	11.9
ResNet101*	44,549,160	83.79	89.40	86.31	88.01	1901.0	11.8
DenseNet121	7,978,856	97.00	96.67	96.67	96.30	453.7	9.4
DenseNet121*	7,978,856	97.10	94.90	95.97	96.96	1357.0	9.0
VGG-19	143,667,240	96.67	96.00	96.34	96.08	499.6	9.4
VGG-19*	143,667,240	93.51	97.37	95.31	96.03	1508.0	13.6
InceptionV3	27,161,264	95.01	95.11	95.06	95.24	390.7	7.4
InceptionV3*	27,161,264	93.42	94.98	94.1	95.1	1044.0	7.7
Classifier 1^*^	**306,467**	97.30	96.87	97.08	97.92	**123.3**	**2.0**
Classifier 2^*^	339,235	97.04	97.08	97.06	97.98	189.9	2.7
Classifier 3^*^	355,619	97.37	97.76	97.57	98.28	165	5.5
TOPCONet	1,001,324	**97.84**	**97.85**	**97.85**	**98.61**	N/A^#^	11.2

For citing recall (Rec), precision (Pre) and F1-score micro-average method is used.

*After the CNN models’ name means that the models have been trained from scratch. ^#^As the proposed ensemble method is non trainable, hence time required to run on the training split is not applicable (N/A).

RA indicates recognition accuracy and bold faced numbers indicate the best scores.

From these tables the number of trainable parameters for the proposed Classifier 1, Classifier 2 and Classifier 3 can be observed as 306,467; 339,235; and 355,619, respectively. Classifier 3 uses the maximum number of trainable parameters (355,619) which is 28.49% of trainable parameters used in the SqueezeNet model (1,248,424), which itself is considered to have the least number of trainable parameters among the well-known deep CNN models such as MobileNetV2 (3,504,872), VGG-19 (143,667,240) and DenseNet121 (7,978,856). Moreover, these tables also show that the total number of trainable parameters used in the proposed TOPCONet model is 1,001,324 (trainable parameters of Classifier 1 + Classifier 2 + Classifier 3 + TOPSIS-aided ensemble) which is evidence that although TOPCONet consists of three base classifiers it uses less number of trainable parameters than the state-of-the-art CNN models. Hence, we can safely comment that the proposed TOPCONet is computationally less expensive than the popularly used state-of-the-art CNN models used in literature in terms of trainable parameters.

For Dataset-1, when compared in terms of accuracy, the proposed Classifier 3 among the three base classifiers demonstrates the highest recognition accuracy which is 0.58% more than the second-highest 98.20% obtained using the VGG-19 model which uses transfer learning concept. In the case of the precision score, the MobileNetV2 model trained from scratch on Dataset-1 offers a better result (98.39%) which is 0.05%, more than our Classifier 2. For recall the DenseNet121 (trained from scratch) and MobileNetV2 (following transfer learning concept) give better results over our base classifiers. In the case of the F1-score, the proposed Classifier 3 performs the best. However, TOPCONet outperforms all the CNN models on this dataset in terms of all the parameters. Now comparing our base classifiers with other popularly used CNN models on Dataset-2, it is evident that the proposed Classifier 3 outperforms all the other popular CNN models in terms of recognition accuracy with a difference of $0.63\%$ with respect to the second-best result given by the present Classifier 2. It is also to be noted that apart from the accuracy, the Classifier 3 performs better in terms of precision, recall and F1-score values which are $0.54\%$, $0.48\%$ and $0.77\%$ better than the second-best method.

Although in some cases the proposed base classifiers are not able to outperform the other CNN models, it is to be kept in mind that it has far fewer of trainable parameters than those other CNN models which makes it easier to train, making it appropriate use in a resource constraint environment. It is evident from the training time column that the proposed base classifiers take far lesser time to train than the other state-of-the-art CNN-based classifiers in the case of both the datasets. In the case of CNN models, training from scratch takes more time than using the transfer learning approach. However, in the case of the proposed classifiers although they are being trained from scratch they take less time than the standard CNN models using transfer learning approach.

In a nutshell, from the experimental results discussed in this subsection and shown in Tables 2 and 3 we can safely comment that (1) the proposed customised CNN model takes much less time to train from scratch as compared to state-of-the-art CNN models (please note Classifier 2 take the same input type (i.e., RGB image) as other state-of-the-art CNN models), (2) our customised CNN model also takes less time as compared to other CNN models when they are trained according to the transfer learning concept, (3) both the customised CNN model-based base classifiers and TOPCONet have less trainable parameters compared to other CNN models, (4) unsurprisingly, TOPCONet outperforms all the models with moderate training and testing time.

### Performance comparison: TOPSIS-aided ensemble method vs. standard ensemble methods

We implemented some standard ensemble methods such as product rule, sum rule, weighted average method and majority voting method. After evaluation on the test sets of Dataset-1 and Dataset-2, the performances of the ensemble methods are shown in Table 4. Here we would like to mention that for implementing the TOPSIS-aided ensemble approach, we have first ranked the base classifiers depending on validation accuracy and then assigned the weights (termed as criteria weights in the TOPSIS method) 0.55, 0.35 and 0.1 chronologically from the best to the worst-performing base classifiers. We set these values experimentally. This parameter tuning is conducted only for Dataset-1 while using the same values for evaluating TOPCONet on Dataset-2. In terms of accuracy, the TOPSIS aided ensemble method outperforms all the other standard ensemble methods used here for comparison on Dataset-1. The difference in accuracy is $0.16\%$ when compared to the second best performers: weighted average and sum rule based ensemble methods. Considering the results on Dataset-2, the difference in accuracy between the highest accuracy obtained using the proposed method and the second best result obtained by product rule ensemble method is $0.19\%$.

**Table 4:** Performance comparison of the TOPSIS aided ensemble method with other popular ensemble methods.

Category	Mmethod	Dataset-1				Dataset-2			
		Precision	Recall	F1-score	Accuracy	Precision	Recall	F1-score	Accuracy
Hard-voting	Majority voting	98.40	97.76	98.42	98.45	96.74	97.68	97.17	98.21
Soft-voting	Product rule	98.34	97.34	98.00	98.37	98.10	97.97	98.04	98.42
	Sum rule	98.65	97.98	98.32	98.62	97.90	97.78	97.85	98.32
	Weighted average	98.65	97.98	98.32	98.62	97.90	97.78	97.84	98.32
TOPSIS-aided		98.47	98.26	98.37	98.78	97.84	97.85	97.85	98.61

All the scores reported are in %.

In terms of precision for Dataset-1, the sum rule and weighted average-based ensemble methods exceed that of the proposed method by $0.18\%$ and for Dataset-2, the product rule-based ensemble outperforms the proposed TOPSIS-aided ensemble method by $0.26\%$. The highest result for performance in terms of the recall value is given by the proposed method which is $98.26\%$ for Dataset-1 but for Dataset-2 it is the product rule based ensemble that exceeds the proposed method by $0.12\%$. If we compare in terms of F1-score on the Dataset-1, it can be concluded that the majority voting ensemble method gives the best F1-score with $0.05\%$ more than the proposed method while for Dataset-2, it is the product rule based ensemble method that gives the highest F1-score with the difference being $0.19\%$ from the proposed method.

Moreover, the performances of the ensemble methods of weighted average and sum rule are almost the same. It may have happened because, in the case of the weighted average method, we take the normalised validation accuracies as the weights for each classifier which themselves are almost similar and so despite using weighted average ensemble to prioritise the best performing classifiers, the ensemble gives almost equal priority to the three classifiers which in turn become similar to sum rule based-ensemble method.

### Analysis of execution time at module level

In this section, we describe the execution time for different steps in TOPCONet with the machine configuration described previously. The time required to process one batch of samples (i.e., 16 images at a time) at different stages is tabulated in Table 5. From Table 5 it is clear that the total time required to test one sample using the proposed approach is $(240+12+13+14+4)/16$ which is nearly 18 ms. The increase in training time for Classifier 2 and Classifier 3 over Classifier 1 is attributed to the fact that the number of trainable parameters for Classifiers 2 and 3 is higher than Classifier 1. This is because Classifier 2 and Classifier 3 accept 3-channel and 4-channel input images respectively, while Classifier 1 accepts 1-channel input images.

**Table 5:** Time required to process 16 sample images at a time. The training time reported is recorded as the sum of total time required to train for each epoch. The time reported is the average of five independent runs.

Process	Time to train (in ms)	Time to test (in ms)
Pre-processing	240	240
Classifier 1	350	12
Classifier 2	375	13
Classifier 3	425	14
TOPSIS ensemble	–	4

### Performance of the TOPCONet model using 5-fold cross validation technique

To test the effectiveness of the proposed model, we also evaluate our model performance using the standard 5-fold cross-validation scheme. In this setting, we split the dataset into five separate folds. Each time sample of four splits is considered a training samples while the rest samples are used for the testing. The detailed experimental outcomes are recorded in Table 6. From Table 6 it is evident that for Dataset-1, the TOPCONet model performs the best on Fold 4 experiment with accuracy 98.24%. In the case of Dataset-2, the highest accuracy for the proposed ensemble method is achieved during Fold 3 experimentation which is 97.85%. For the base classifiers in the case of Dataset-1, the highest accuracy for Classifier 1 is achieved in Fold 2, for Classifier 2 it is Fold 4 and for Classifier 3 it is Fold 3 experiment. For Dataset-2, during Fold 3 experimentation we attain the highest accuracy for all the base classifiers.

**Table 6:** Accuracies (in %) while evaluating using 5-fold cross validation scheme.

Fold	Dataset-1				Dataset-2			
	Classifier 1	Classifier 2	Classifier 3	Ensemble	Classifier 1	Classifier 2	Classifier 3	Ensemble
Fold1	96.09	96.42	95.84	96.58	96.43	96.46	95.84	96.83
Fold2	97.79	97.74	97.55	98.12	95.64	95.60	95.80	96.27
Fold3	97.02	96.58	97.72	97.80	97.46	97.52	97.72	97.85
Fold4	97.71	97.79	97.71	98.24	96.73	96.86	96.93	96.96
Fold5	96.57	96.74	96.82	97.06	95.59	95.95	95.16	96.13
Maximum	97.79	97.79	97.72	98.24	97.46	97.52	97.72	97.85
Minimum	96.09	96.42	95.84	96.58	95.59	95.60	95.16	96.13
Average	97.04	97.05	97.13	97.56	96.37	96.48	96.29	96.80
SD	0.65	0.59	0.72	0.64	0.70	0.67	0.91	0.61

### Performance comparison: proposed method (TOPCONet) vs. state-of-the-art methods

To test the effectiveness of the proposed TOPCONet model, we compare its performance with the performances of some state-of-the-art methods proposed by Khan et al.^36^, Jain et al.^57^, Hussain et al.^37^, Ismael et al.^58^, Das et al.^25^, Goel et al.^38^, and Paul et al.^24^ on Dataset-1 while for Dataset-2 we compare the methods proposed by Aslan et al.^39^, Ouchicha et al.^29^, Kedia et al.^59^, Ahmad et al.^60^, Chowdhury et al.^56^, Sedik et al.^18^, Wu et al.^30^, Panetta et al.^20^, Yang et al.^61^, Paul et al.^24^, Gour et al.^33^, Gour et al.^26^, Hasoon et al.^27^, Bashar et al.^34^, Senan et al.^35^, Naeem et al.^40^, Goyal et al.^41^, Roy et al.^28^ and Senan et al.^35^. To have a fair comparison with these methods include the experimental setups, especially the approach followed to prepare the samples of train and test sets, used by these methods. We found that two major approaches were used by these researchers: (1) partitioning the samples of the entire dataset into train and test sets^18,20,25,34–36,38,39,57–59,61^, as shown in Table 1 (we call this experimental setup as hold-out test set approach) and (2) standard 5-fold cross validation approach^26,27,29,30,33,37,40,41,56,60^ as described in subsection “Performance of TOPCONet model using 5-fold cross validation technique”. However, the method designed by Paul et al.^24^ do not follow any of the mentioned experimental setups and hence we have developed the models at our end and evaluated following our experimental schemes. For the method proposed by Paul et al.^24^ we use transfer learning concept. Likewise, we also estimate the performances of the proposed TOPCONet model using both the mentioned approaches. On the other hand, the method proposed by Bashar et al.^34^ and Senan et al.^35^ designed for 4-class classification problem. Hence, for the comparison with our method we train and test these two models on the present datasets using their experimental setups, and consider the best performance obtained. The comparative results are recorded in Table 7 and Table 8 for Dataset-1 and Dataset-2 respectively.

**Table 7:** Performance comparison of the proposed method with some state-of-the-art models on the Dataset-1.

Work Ref.	Technique	Experimental protocol	Performance (in %) in terms of			
			Precision	Recall	F1-score	Accuracy
Khan et al.^36^	CoroNet	Hold-out test set	95.00	96.90	95.94	95.00
Jain et al.^57^	Xception	Hold-out test set	98.00	94.60	96.00	97.00
Hussain et al.^37^	CoroDet	5-Fold cross validation	96.34	96.00	96.00	96.66
Ismael et al.^58^	End to end CNN	Hold-out test set	95.67	94.67	95.00	96.09
Das et al.^25^	Bi level prediction	Hold-out test set	97.87	98.14	98.00	98.45
Goel et al.^38^	OptCoNet	Hold-out test set	92.88	96.25	95.25	97.78
Paul et al.^24^	Inverted bell ensemble	Hold-out test set	97.21	97.81	97.50	97.97
Paul et al.^24^	Inverted bell ensemble	5-Fold cross validation	97.12	97.62	97.39	97.85
Gour et al.^33^	UA-ConvNet model	5-Fold cross validation	98.49	98.26	98.36	98.09
Gour et al.^26^	Stacked CNN model	5-Fold cross validation	97.62	98.52	97.50	97.27
Hasoon et al.^27^	LBP-KNN model	5-Fold cross validation	97.80	100.0	98.88	98.70
Bashar et al.^34^	Optimized CNN model	Hold-out test set	95.67	93.34	94.67	95.20
Senan et al.^35^	ResNet50	Hold-out test set	98.00	98.67	98.67	98.70
Naeem et al.^40^	CNN-LSTM model	Hold-out test set	95.00	95.00	95.00	96.60
Goyal et al.^41^	F-RRN-LSTM model	Hold-out test set	88.89	95.41	92.03	94.31
Proposed	TOPCONet	Hold-out test set	98.67	98.00	98.34	98.78
		5-Fold cross validation	98.10	97.53	97.81	98.24

**Table 8:** Performance comparison of the proposed method with some state-of-the-art models on the Dataset-2.

Work Ref.	Technique	Experimental protocol	Performance (in %) in terms of			
			Precision	Recall	F1-score	Accuracy
Aslan et al.^39^	mAlexNet+BiLSTM	Hold-out test set	98.77	98.76	98.76	98.70
Aslan et al.^39^	mAlexNet	Hold-out test set	98.16	98.26	98.20	98.14
Ouchicha et al.^29^	CVDNet	5-Fold cross validation	96.72	96.84	96.68	96.69
Kedia et al.^59^	CoVNet-19	Hold-out test set	98.34	98.34	98.34	98.20
Ahmad et al.^60^	InceptionV3+MobileNetV2	5-Fold cross validation	97.56	97.54	97.55	98.77
Chowdhury et al.^56^	CheXNet	5-Fold cross validation	96.61	96.61	96.61	97.74
Sedik et al.^18^	ConvLSTM	Hold-out test set	94.67	97.09	95.64	95.96
Wu et al.^30^	ULNet	5-Fold cross validation	96.93	96.60	96.60	95.25
Panetta et al.^20^	Classical Fibonacci p-pattern	Hold-out test set	97.78	96.90	97.32	97.79
Panetta et al.^20^	Shape dependent Fibonacci p-pattern	Hold-out test set	97.20	96.76	96.69	98.03
Yang et al.^61^	Fast.AI ResNet	Hold-out test set	97.00	97.00	97.00	97.00
Paul et al.^24^	Inverted bell ensemble	Hold-out test set	97.24	97.25	97.24	97.64
Paul et al.^24^	Inverted bell ensemble	5-Fold cross validation	96.84	96.72	96.72	97.12
Gour et al.^33^	UA-ConvNet model	5-Fold cross validation	99.51	98.00	98.73	98.90
Roy et al.^28^	CoWarriorNet	Hold-out test set	94.66	91.33	92.66	97.80
Bashar et al.^34^	Optimized CNN model	Hold-out test set	97.00	93.67	95.67	96.55
Senan et al.^35^	ResNet50 model	Hold-out test set	97.00	97.67	97.67	98.01
Goyal & Singh^41^	F-RRN-LSTM model	Hold-out test set	93.65	96.78	95.19	95.04
Proposed	TOPCONet	Hold-out test set	97.84	97.85	97.85	98.61
		5-Fold cross validation	96.99	96.99	96.99	97.85

For Dataset-1, among the state-of-the-art methods evaluated following the hold-out test set approach, the methods proposed by Senan et al.^35^ and Das et al.^25^ stand first and second with a test accuracy of 98.70% and 98.45% respectively. Also, the best recall, F1-score and precision values are given by the optimized ResNet50 model by Senan et al.^35^ with values 98..67%, 98.67% and 98.00% respectively. Whereas, the pre-trained Xception model proposed by Jain et al.^57^ gives the best precision of 98.00% among the state-of-the-art methods. Now, when comparing TOPCONet with state-of-the-art methods that followed the hold-out test set based experimental setup, results from Table 7 are the evidence that the highest accuracy for the Dataset-1 is given by the proposed method, which is $0.08\%$ more than the optimized ResNet50 model proposed by Senan et al.^35^. In terms of precision score, our method exceeds the pre-trained Xception model by Jain et al.^57^ and the optimized ResNet50 model by Senan et al.^35^ by $0.67\%$. However, in terms of F1-score the optimized ResNet50 model outperforms our TOPCONet method by ($0.33\%$.) and in terms of recall it is outperformed by Senan et al.^35^ by $0.67\%$. In the case of methods that followed the five-fold cross-validation approach, the proposed method outperforms the state-of-the-art method that employed CoroDet.

For Dataset-2 among the state-of-the-art methods that followed five-fold cross-validation experimental setup, the best performer is the UA-ConvNet model which is proposed by Gour et al.^33^ with an accuracy of $98.90\%$ while the second-best accuracy is provided by the ensemble of InceptionV3 and MobileNetV2 proposed by Ahmad et al.^60^ with a value of $98.77\%$. In the case of precision, recall and F1-score the ensemble of InceptionV3 and MobileNetV2^60^ outperforms all other methods in the case of 5-fold cross-validation experimental setup. It also outperforms the proposed method with 5-Fold cross-validation by $0.57\%$, $0.55\%$, $0.56\%$ and $0.92\%$ in precision, recall, F1-score and recognition accuracy respectively.

When it comes to the comparison of the methods using the hold-out data splitting method, the best accuracy, precision, recall and F1-score are achieved by the hybrid architecture using the modified AlexNet model and BiLSTM with the value of $98.70\%$, $98.77\%$, $98.76\%$ and $98.76\%$ respectively. However, this method outperforms the proposed method in precision, recall, F1-score and accuracy by $0.93\%$, $0.91\%$, $0.91\%$ and $0.09\%$ respectively. Although from the comparison, it can be observed that some methods have outperformed the proposed method in Dataset-2 for both the hold-out test set and five-fold cross-validation experimental setups but it is to be kept in mind that the number of parameters used by the proposed method is far less than that of these state-of-the-art methods. The total number of parameters of the combination of three classifiers is around 1 million which is far less than that of AlexNet, MobileNetV2 and InceptionV3 models as shown in Table 2.

### Performance of TOPCONet on COVIDx CXR-3 dataset

To investigate scalability and robustness of the proposed TOPCONet model, we have evaluated its performance on a recently published large daraset, known as COVIDx CXR-3 Dataset^62^. This dataset was made public by Wang et al.^17^. It consists of 30,000 chest X-ray images from over 16,490 subjects (either COVID-19 positive patients or normal subjects). The images in the dataset are already partitioned into train and test sets by the researchers in the work^17^. In the train set of the dataset, there are 16,400 chest X-rays of COVID-19 positive patients and 13,992 chest X-rays belonging to non COVID-19 subjects whereas, the test set contains 200 chest X-ray images of the COVID-19 positive and negative patients each. We have followed the standard dataset divisions to conduct our experiments. The performance of the TOPCONet along with the base classifiers are provided in Table 9. In order to compare the performances of our model on this dataset, we compare it with some state-of-the-art COVID-19 detection methods like Xception model used by Jain et al.^57^, the CheXNet model by Chowdhury et al.^56^, the ResNet50 model used Senan et al.^35^, the CovidConvLSTM model by Dey et al.^19^ and the use of VGG-16 as a transfer learning model that was used by Bashar et al.^34^. Performances of these state-of-the-art models are also recorded in Table 9 along with performances of our final model and base classifiers. From Table 9, it can be concluded that the proposed TOPCONet model gives the highest performance in all metrics like accuracy, precision, recall and F1-score. The second and third highest results in all metrics are given by Classifier 1 and Classifier 2 respectively. However, the Classifier 3 is outperformed in terms of accuracy by other state-of-the-art methods. Apart from the performance, present models acquire less trainable parameters than the past methods used here for comparison.

**Table 9:** Performances of TOPCONet model and its sub-modules along with some state-of-the-art models on COVIDx Dataset.

Work Ref.	Technique	#Trainable parameters	Performance (in %) in terms of			
			Precision	Recall	F1-score	Accuracy
Jain et al.^57^	Xception	22,910,480	79.50	74.00	73.00	74.25
Chowdhury et al.^56^	CheXNet	20,242,984	82.50	75.00	73.50	74.75
Bashar et al.^34^	Optimized CNN	138,357,544	87.50	85.50	85.00	85.50
Senan et al.^35^	ResNet50	25,636,712	80.50	77.00	76.50	77.25
Dey et al.^19^	CovidConvLSTM	363,996,809	88.71	86.75	86.58	86.75
Proposed	Classifier 1	306,467	94.00	92.61	93.30	93.25
Proposed	Classifier 2	339,235	92.00	92.46	92.23	92.25
Proposed	Classifier 3	355,619	81.00	69.53	74.83	72.75
Proposed	**TOPCONet**	1,001,324	**94.50**	**94.03**	**94.26**	**94.25**

The best values are highlighted in bold.

## Discussion

It has already been mentioned that in this work, we propose a new CAD system (i.e., TOPCONet) for COVID-19 and pneumonia detection from chest X-ray images. The TOPCONet model is computationally inexpensive and efficient. The performance of the model is evaluated on two publicly available datasets and the obtained results are described in the previous section. However, in this section, we discuss more findings of the TOPCONet model. To start with we discuss the performance and the number of training parameters of all the basic components of the model. It is to be noted here that TOPCONet comprises of four major components: three base classifiers, namely Classifier 1, Classifier 2 and Classifier 3 and TOPSIS aided ensemble process. The performances on Dataset-1 and Dataset-2 using the experimental setup are discussed in the subsection “Dataset description” and the number of trainable/tunable parameters present in these major components are provided in Table 2. From the performances recorded in Tables 2 and 3 we can see that Classifier 3 that accepts 4-channel input images performs the best among the base classifiers for both datasets. From this observation, we can safely comment that combining the edge image, generated by Robert's edge detection technique, with the original image helps us to obtain better performance. From these results, we can also say that our model learns the association of features.

**Figure 6:**
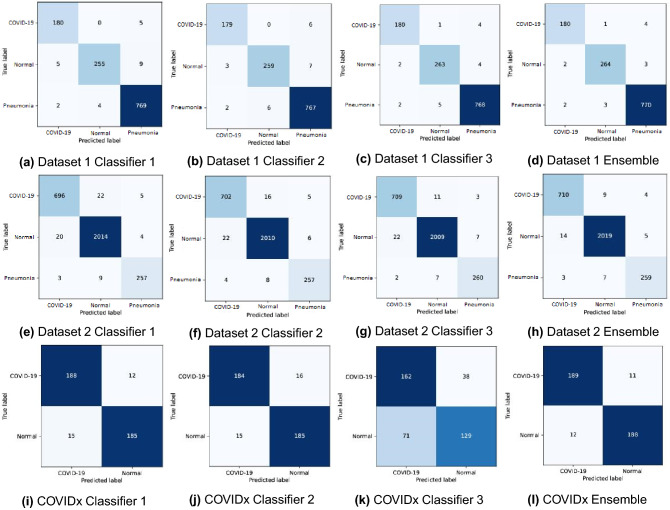
Confusion matrices obtained on Dataset-1, Dataset-2 and COVIDx dataset.

In addition to these, we can also observe that after the ensemble of the outputs from the three base classifiers using the TOPSIS-aided ensemble method, it results in an increase of accuracy by $0.25\%$ for Dataset-1 and in the case of Dataset-2 the increase in accuracy is $0.33\%$. The results establish that the TOPSIS-aided ensemble method can learn the better association of confidence scores provided by the classifiers. For a deeper understanding, we examine the confusion matrices of the base classifiers and the TOPSIS aided ensemble method for all the datasets. The confusion matrices are shown in Fig. 6. From the figure, we can conlcude the TOPSIS aided ensemble works optimally for all the three datasets. Hence we can also claim that the proposed method works well across the datasets. The proposed ensemble method is able to capture multiple complimentary information provided by the base classifiers for COVID-19 and pneumonia detection.

We can also observe from Tables 2 and 3 that a certain number of parameters are trained for each of the base classifiers. This can be explained because the input dimension of each of the base classifiers differs. Moreover, it must be noted that the ensemble approach consists of several criteria (in this case, three). Corresponding to each of the criteria, a criteria weight is assigned. These weights are manually tuned i.e., these are not trained by any learning methods.

**Figure 7:**
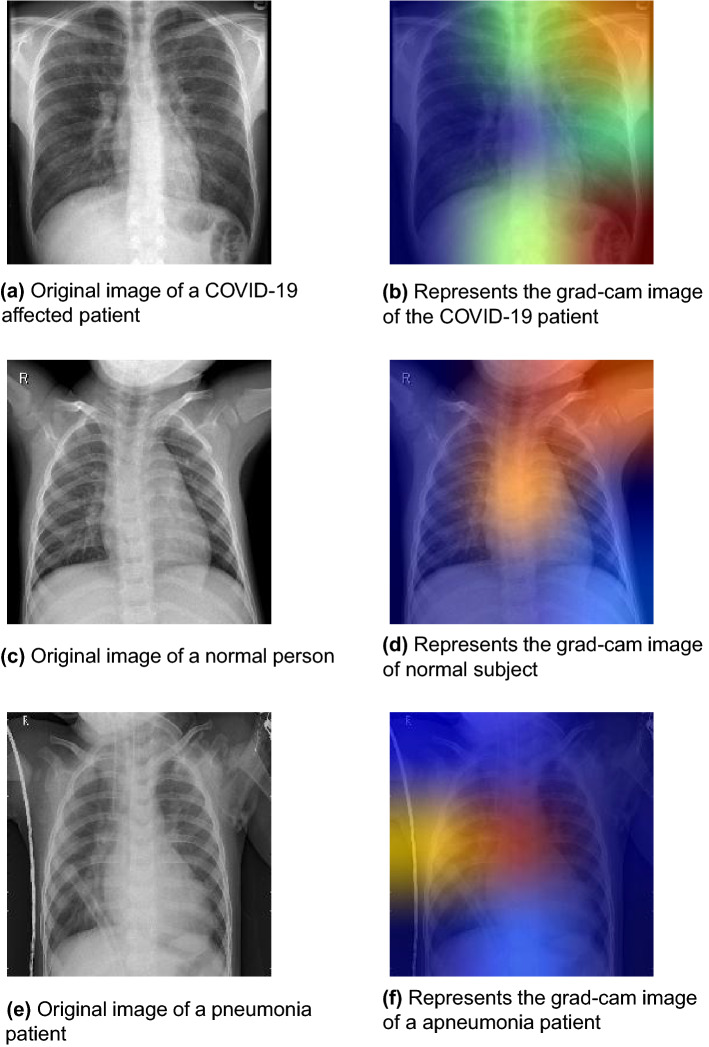
Grad-cam images along with original chest X-ray images for the base classifier 2 trained on the Dataset-1. Images are taken from the public datasets found in^51,52^.

For further analysis of the proposed 3-channel CNN model (i.e., Classifier 2), we use grad-cam analysis, which works by using the gradients generated inside the model to create a heat-map to show the regions where the model focuses to perform the intended classification task. In Fig. 7, the original chest X-ray images along with their grad-cam counterparts are shown. In this figures, we observe that the TOPCONet focuses mainly on the lung area. It is noteworthy that a few activation regions can be seen outside the lung. This is probably because the model learns a few redundant features along with the useful ones. These redundant features are the ones that get insignificant importance from the classifier (FCNNs in this case) during the final classification.

### Advantages and limitations of the proposed method

The advantages of the proposed model are listed below:

- The proposed CNN model can learn effective features from input images of different types.
- It has fewer tuning parameters compared to state-of-the-art CNN models.
- The TOPSIS aided ensemble method can efficiently aggregate the confidence scores estimated by the base classifiers to provide the final class.
- The ensemble method used here has very few tunable parameters (same as the number of base classifiers used in ensemble mechanism) and hence very little additional cost is required.

To provide our observations on the proposed work, we also mention some of its limitations which are as follows:

- The proposed CNN model might not work well on rotated and arbitrarily cropped images. It might require different augmented training samples like other CNN models.
- The TOPSIS aided ensemble method fails to classify a sample if two of the base classifiers incorrectly classify it with high confidence.
- As we have used deep learning models as our base learners, it demands resources such as graphics processing unit (GPU), dedicated random access memory (RAM) etc. for training the same.

## Conclusion

Keeping in mind the present scenario of COVID-19 cases, the situation needs to be addressed properly to help humanity to come out of this crisis as soon as possible. In the present work, we propose a CAD system, called TOPCONet, for screening COVID-19 and pneumonia which performs well on publicly available three chest X-ray datasets. In TOPCONet, we first pre-process the images to obtain different forms of the original images, which are further used to train and evaluate the model. The skeletal structure of the base classifiers of the ensemble approach is the same, however, the type of input differs for each of the cases. We combine the decisions of these base classifiers using the proposed TOPSIS aided ensemble method. The proposed model can learn deep features better, and it performs at par with the state-of-the-art methods. It is noteworthy to mention that our customised CNN model uses a very less number of trainable parameters compared to the popularly used CNN models in several deep learning-aided CAD systems. It should be noted that these datasets suffer from the problem of imbalanced classes. Hence further works can be focused on handling this issue efficiently. Since our model gives an end-to-end classification result for COVID-19 and pneumonia diseases using chest X-ray images and is lighter in nature, it may be employed in detecting such diseases to ease the burden on radiologists and doctors. However, the proper advice of medical professionals should be considered along with the outcome from this model. Apart from these, further work may be focused on using domain knowledge as it may increase the model performance. Last but not the least, the present model can be used to extract features first and then a suitable feature selection technique may be employed to select a near-optimal subset of features that may further reduce the storage and time requirements.
